# Evaluation of the long-term treatment effects of intravenous idursulfase in patients with mucopolysaccharidosis II (MPS II) using statistical modeling: data from the Hunter Outcome Survey (HOS)

**DOI:** 10.1186/s13023-021-02052-4

**Published:** 2021-10-30

**Authors:** Joseph Muenzer, Jaco Botha, Paul Harmatz, Roberto Giugliani, Christoph Kampmann, Barbara K. Burton

**Affiliations:** 1 University of North Carolina at Chapel Hill, 101 Manning Drive CB# 7487, Medical School Wing E Room 117, Chapel Hill, NC 27599-7487 USA; 2 Takeda Pharmaceuticals International AG, Zurich, Switzerland; 3 UCSF Benioff Children’s Hospital Oakland, Oakland, CA USA; 4 Department of Genetics, UFRGS, Medical Genetics Service, HCPA, and INAGEMP, Porto Alegre, Brazil; 5 Johannes Gutenberg University, Mainz, Germany; 6 Ann & Robert H. Lurie Children’s Hospital of Chicago, Chicago, IL USA

**Keywords:** Mucopolysaccharidosis II, MPS II, Hunter syndrome, Lysosomal storage disease, Statistical modeling, Disease registry, Idursulfase, Enzyme replacement therapy

## Abstract

**Background:**

Mucopolysaccharidosis II (MPS II; Hunter syndrome) is a rare, life-limiting lysosomal storage disease caused by deficient iduronate-2-sulfatase activity. Enzyme replacement therapy (ERT) with intravenous (IV) idursulfase can stabilize or improve many somatic manifestations, but there remains a need for further analysis of long-term treatment outcomes. Using data from patients with MPS II enrolled in the Hunter Outcome Survey (HOS), mixed modeling was performed to evaluate and predict the effects of IV idursulfase treatment on selected clinical parameters for up to 8 years following treatment start. The modeling population comprised male patients followed prospectively in HOS who had received IV idursulfase for at least 5 years and who had data available for two or more time points (at least one post-ERT). Age at ERT start and time since ERT start were included as covariates.

**Results:**

In total, 481 patients were eligible for inclusion in at least one model. At 8 years post-ERT start, improvement from baseline was predicted for each age group (< 18 months, 18 months to < 5 years and ≥ 5 years at treatment start) in the following parameters: mean urinary glycosaminoglycan levels (percentage changes of > –75% in each group), mean left ventricular mass index (decreases of ~ 1 g/m^2^) and mean palpable liver size (decreases of > 2 cm). Improvements in mean 6-min walk test distance (increase of > 50 m) and stabilization in percent predicted forced vital capacity and forced expiratory volume in 1 s (decreases of ~ 4 and ~ 9 percentage points, respectively) at 8 years post-ERT start were predicted for patients aged ≥ 5 years at ERT start (these assessments are unsuitable for patients aged < 5 years). Predicted changes over time were similar across the three age groups; however, overall outcomes were most favorable in children aged < 18 months at ERT start.

**Conclusions:**

These findings suggest that the previously reported positive effects of IV idursulfase on the somatic manifestations of MPS II are predicted to be maintained for at least 8 years following ERT initiation and highlight the value of statistical modeling to predict long-term treatment outcomes in patients with rare diseases.

**Supplementary Information:**

The online version contains supplementary material available at 10.1186/s13023-021-02052-4.

## Background

Mucopolysaccharidosis II (MPS II; Hunter syndrome; OMIM 309900) is a rare, X-linked lysosomal storage disease (LSD) with an estimated birth prevalence of 0.10–2.16 per 100,000 live births [1]. The disease is caused by deficient activity of the lysosomal enzyme iduronate-2-sulfatase (I2S), which leads to the accumulation of glycosaminoglycans (GAGs) throughout the body and results in progressive, multisystemic clinical signs and symptoms [2, 3].

Although somatic manifestations are apparent in all patients with MPS II, clinical presentation and disease severity vary [2]. Approximately two-thirds of patients have the neuronopathic form of the disease, with central nervous system (CNS) involvement and cognitive impairment [2–5]. Patients with the non-neuronopathic form of MPS II may survive into their fifth or sixth decade, with respiratory and/or cardiac complications being two of the most common causes of death [2, 6, 7]. Patients with CNS involvement tend to experience a more rapid disease progression and die in the second decade of life, typically with respiratory and/or cardiac complications in addition to severe neurological involvement [2, 6, 7].

Owing to the multisystemic clinical presentation of MPS II, effective management of patients generally requires a multidisciplinary approach across a range of specialties [8]. Disease-specific treatment for MPS II is available in the form of intravenous (IV) enzyme replacement therapy (ERT) with recombinant human I2S. IV ERT with idursulfase (Elaprase®, Shire [a Takeda company], Lexington, MA, USA) has been available since 2005 and has been shown to be well tolerated and to stabilize or improve a range of clinical parameters [9]. The effects observed following treatment with IV idursulfase include increased forced vital capacity (FVC) and forced expiratory volume in 1 s (FEV_1_), stabilized left ventricular mass index (LVMI), increased distance walked in the 6-min walk test (6MWT), increased joint range of motion in the shoulder, decreased liver and spleen sizes and decreased urinary glycosaminoglycan (uGAG) levels, with these trends observed both in clinical trials and analyses of real-world data [9–16]. In addition, analyses of real-world data have demonstrated increased survival in patients with MPS II who received IV idursulfase compared with untreated patients [7, 16].

A previous registry-based analysis has demonstrated ERT-associated clinical improvements in real-world settings at up to 3 years after initiation of IV idursulfase treatment [11], supporting observations from the initial clinical trials [10, 12]. However, many patients with MPS II have now been receiving treatment with IV idursulfase for over 5 years and there is a need to improve our understanding of the long-term outcomes for patients after extended periods of treatment. Although some studies have evaluated outcomes for longer periods, including 10 years of follow-up, these were generally small-scale with geographically limited populations (mostly single-center studies) and the clinical parameters studied varied [13–16]. It is also important to explore the influence of variables such as the age at treatment initiation on overall outcomes because data in this area remain limited.

It remains challenging to collect long-term data and to assess the impact of treatment in a broad population for rare diseases such as MPS II. Disease registries allow the collection and evaluation of data in broader, real-world populations and over a longer time period compared with clinical trials. Combining real-world data from disease registries with statistical modeling methods has the additional advantages of overcoming the problems of missing values and of potential confounding factors, such as appropriate accounting for the time since ERT start, and may therefore offer a more effective approach for evaluating and predicting long-term outcomes. The Hunter Outcome Survey (HOS; NCT03292887) is a large, multicenter, observational registry funded by Shire (a Takeda company) that collects real-world data on the natural history of MPS II and treatment with IV idursulfase [3, 17]. Using global real-world data from a broad population of patients in HOS, we developed statistical models to evaluate and predict the effects of IV idursulfase treatment on selected clinical parameters for up to 8 years following treatment start.

## Methods

### HOS registry design

HOS is designed to collect data on patients with MPS II that are obtained during routine visits and assessments [3]. Patients with MPS II who are untreated or receiving treatment with IV idursulfase are eligible for enrollment; those receiving other forms of ERT or enrolled in an interventional clinical trial are excluded. Patients who have undergone a bone marrow transplant (BMT) are also eligible to enroll in the registry. Data can be included from patients who were alive at enrollment (prospective patients) or, if local regulations permit, who died before enrollment (retrospective patients).

A wide range of clinical and demographic data are captured in the registry based on investigations performed at each clinic; there are no predetermined assessments. The presence of cognitive impairment is recorded in HOS based on the answer to the following question: ‘Cognitive impairment? Yes/No’; this may reflect the results of standardized cognitive tests or the clinical impression of the treating physician.

Independent Review Board/Ethics Committee approval was obtained for all participating centers before patient enrollment; written informed consent was also obtained from each patient, their parents or a legal representative.

### Patient population

Male patients followed prospectively in HOS who had received IV idursulfase for at least 5 years were included in the modeling population. Only patients with a confirmed signed informed consent form and who had information available for two or more time points (at least one post-ERT) for the relevant parameter were included. Those who had undergone a BMT and/or been previously enrolled in a clinical trial for idursulfase were excluded from this analysis because it was considered that their inclusion may confound interpretation of the effects of ERT.

### Clinical parameter measurements

uGAG levels recorded in the registry were obtained from either a local or a central laboratory and were measured using the standard methods available at each laboratory. Percentage changes from pre-ERT levels were calculated to allow comparison of trends among measurements performed using different methods. Patients without a baseline pre-ERT uGAG measurement (recorded within the year immediately before the first dose of IV idursulfase) were excluded from the analysis of percentage change from baseline. uGAG values above 1500 µg/mg (169.5 mg/mmol) creatinine were considered to be unreliable and were excluded.

LVMI was calculated from an echocardiogram using the Devereux formula [18] and adjusted for body surface area (BSA) using height or weight measurements taken during the 3 months before or after the assessment date. LVMI values above 400 g/m^2^ were considered to be unreliable and were excluded from the model. Models were also developed to include or exclude patients with left ventricular hypertrophy (LVH) at baseline, defined as LVMI > 102 g/m^2^ [19].

FVC and FEV_1_ were expressed as both absolute values and percent predicted values, with reference to the expected value in an individual without MPS II of the same age, sex and height [20, 21]. FVC and FEV_1_ measurements corresponding to absolute values above 10 L were considered unreliable and excluded from the model. For FVC, FEV_1_ and 6MWT models, only data from patients aged at least 5 years at the date of assessment were included because these assessments are unsuitable for very young patients [17]. Patients who had cognitive impairment at any time were also excluded from these models for the same reason. In addition, for the 6MWT model only, assessments performed with assistance were excluded.

Liver size was determined by palpation according to standard clinical practice at each center [22]. For any patient whose liver was no longer reported as palpable, liver size was imputed as zero in the model. Palpable liver sizes above 15 cm were considered to be unreliable and were excluded from the model.

For all parameters, if more than one pre-ERT value within the year was available, the most recent was used as the baseline value. Measurements recorded more than 8 years after the first dose of ERT were also excluded. The group of patients with post-ERT data available may not be the same as the group with baseline data available owing to the nature of long-term follow-up, with the frequency of routine clinic visits differing between patients, and the fact that some patients enrolled in HOS after starting ERT. Patients were not required to have a baseline measurement available for the relevant parameter to be included. Stated differences between clinical parameter values from pre-ERT to 8 years post-ERT start are therefore indicative of the general trend only and should not be considered as a measurement of change from baseline.

### Statistical modeling

A random coefficients mixed model was used, with subject and time (day) of assessment as random effects and age group at ERT start (0 to < 18 months, 18 months to < 5 years and ≥ 5 years) as a fixed effect. An unstructured covariance matrix was used, with the Kenward–Roger method to adjust the degrees of freedom. All analyses were performed based on data collected in HOS up to January 23, 2020. Treatment effect was evaluated within each age group, with values predicted for clinical parameters at baseline (pre-ERT) and at yearly intervals for up to 8 years post-treatment. Eight years was selected as the cutoff because this was the latest time point at which the amount of available data was considered sufficient for analysis across all parameters. For FVC, FEV_1_ and 6MWT models, pre-ERT values were deemed ‘not applicable’ for assessments in patients aged under 5 years at ERT start owing to the unsuitability of these assessments for very young children [17]; however, post-ERT values were modeled in these patients from the point at which they reached the age of 5 years.

All data are presented as predicted means (95% confidence interval [CI]). An additional analysis was also conducted to provide an internal validation of the model and to assess robustness, using information from patients who had data for five or more time points. Values that are ‘not determined’ indicate that no patients met the criteria for the parameter and age group in question.

In addition, alternative modeling approaches were investigated to test model assumptions. For the 6MWT model, a log-transformation approach was investigated; however, similar results were obtained overall, and these additional data are not presented. Palpable liver size was also analyzed using a two-part hurdle Poisson mixed-effects model with intercept and time in the study as random effects and ERT starting age group (< 5 years and ≥ 5 years) as a fixed effect.

## Results

### Patient population

In total, 481 of the 1322 patients enrolled in HOS were eligible for inclusion in the statistical model for at least one clinical parameter (Additional file 1; Table S1). These patients had a median age (10th, 90th percentiles) at symptom onset and at diagnosis of 1.5 (0.3, 4.2) years and 3.3 (1.0, 6.9) years, respectively; median age at treatment start was 5.5 (2.0, 17.4) years. Patients were included from 29 different countries in four continents, with just over half (n = 277; 57.6%) from Europe. Cognitive impairment at any time was reported for 319 patients (66.6%) included in the model for at least one clinical parameter.

### uGAG levels

**Fig. 1:**
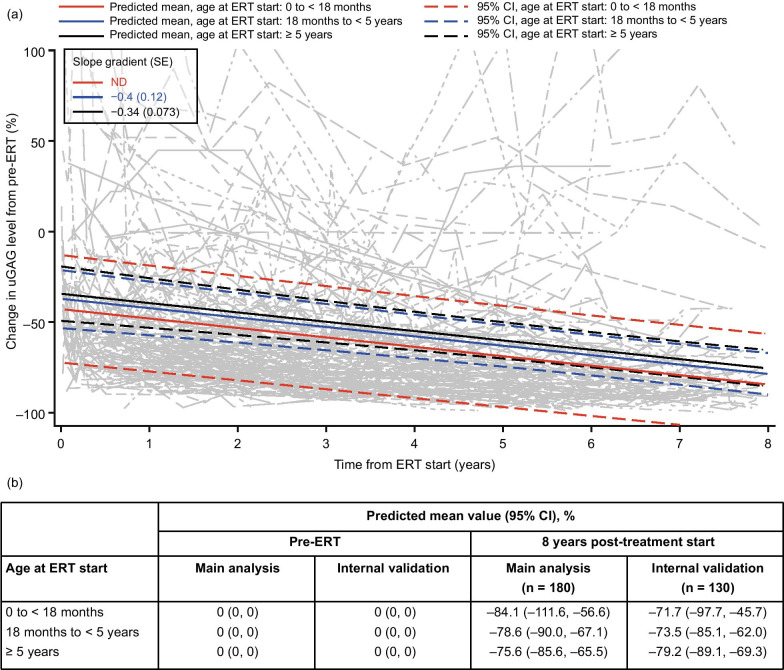
Modeling of percentage change in uGAG levels from pre-ERT over time using data from patients who received ERT with IV idursulfase for 5 years or more. (**a**) Change in predicted means and 95% CIs by age at ERT start (colored lines) and individual patient values (gray lines) from pre-ERT up to 8 years after IV idursulfase start in the main analysis population (patients with data available for at least two time points in total and at least one post-ERT time point; n = 180). The y-axis was terminated at 100% for clarity; some patients had individual values of up to 297% at certain time points, which are not shown here. (**b**) Predicted means by age at ERT start at pre-ERT and 8 years post-ERT in the main analysis population and the internal validation population (patients with data available at five or more time points). Normalized uGAG levels above 1500 µg/mg (169.5 mg/mmol) creatinine were excluded from the model. Abbreviations: CI, confidence interval; ERT, enzyme replacement therapy; IV, intravenous; ND, not determined; SE, standard error; uGAG, urinary glycosaminoglycan

For the 180 patients included in the uGAG analysis, the model-predicted mean uGAG levels continually decreased over time following treatment with IV idursulfase (Fig. 1). Similar decreases were observed across all ages at treatment start, with mean percentage changes of –84.1%, –78.6% and –75.6% from baseline at 8 years post-ERT for patients aged 0 to < 18 months, 18 months to < 5 years and ≥ 5 years at treatment start, respectively (Fig. 1).

### LVMI

**Fig. 2:**
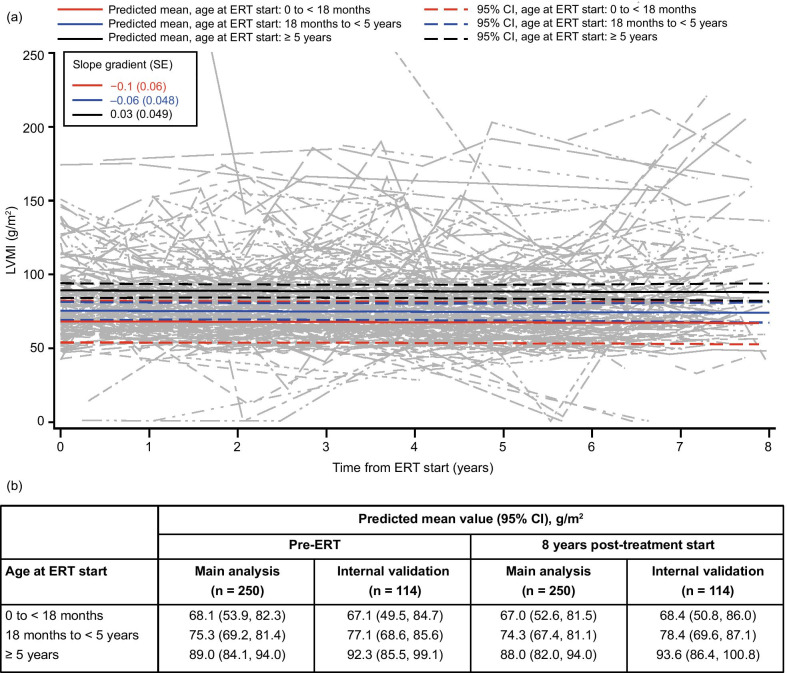
Modeling of LVMI over time following IV idursulfase treatment for patients irrespective of LVH status of baseline using data from patients who received ERT for 5 years or more. (**a**) Change in predicted means and 95% CIs (colored lines) and individual patient values (gray lines) by age at ERT start from pre-ERT up to 8 years after IV idursulfase start in the main analysis population (patients with data available for at least two time points in total and at least one post-ERT time point; n = 250). The y-axis was terminated at 250 g/m^2^ for clarity; some patients had individual values of up to 314 g/m^2^ at certain time points, which are not shown here. (**b**) Predicted means by age at ERT start at pre-ERT and 8 years post-ERT in the main analysis population and the internal validation population (patients with data available at five or more time points). LVMI values > 400 g/m^2^ were excluded from the model. For reference, 50–102 g/m^2^ (indexed to BSA) has previously been defined as a normal LVMI range in male patients [19]. Abbreviations: BSA, body surface area; CI, confidence interval; ERT, enzyme replacement therapy; IV, intravenous; LVH, left ventricular hypertrophy; LVMI, left ventricular mass index; SE, standard error

In the model of LVMI in the overall patient population (including patients both with and without LVH at baseline; n = 250), LVMI remained stable for up to 8 years post-ERT start in all age groups, with decreases of approximately 1 g/m^2^ at 8 years post-ERT compared with baseline across all ages at treatment start (Fig. 2).

A larger decrease in LVMI was observed when the model included only patients with LVH at baseline (n = 19), with decreases of more than 40 g/m^2^ in the mean predicted values at 8 years post-ERT compared with baseline for patients aged 18 months to < 5 years and ≥ 5 years at treatment start (Additional File 1; Fig. S1). It was not possible to model the change over time in patients with LVH aged 0 to < 18 months at ERT start owing to a lack of available data. Evaluation of baseline LVH as a covariate in the model demonstrated that the larger decrease from baseline in LVMI in patients with versus those without LVH at baseline was statistically significant (*p* < 0.0001).

### FVC and FEV_1_

**Fig. 3:**
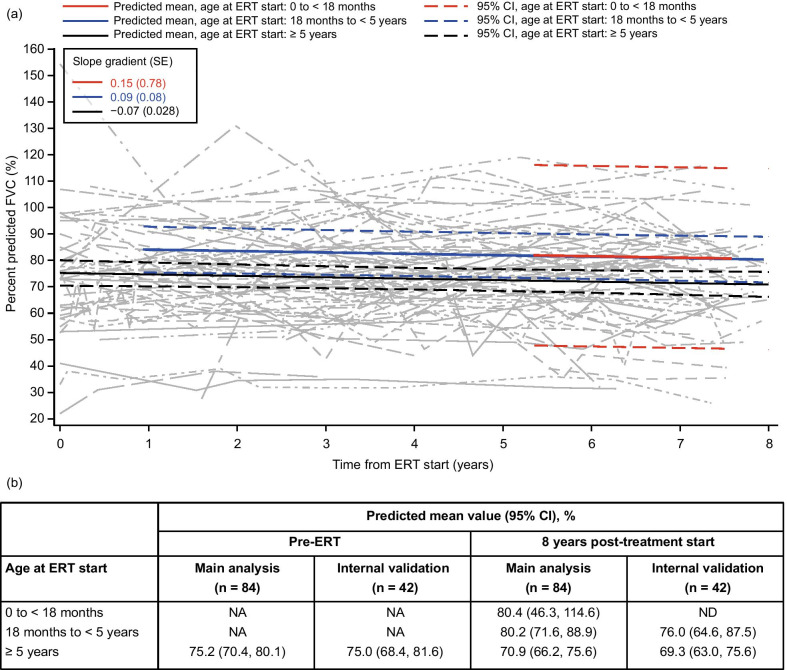
Modeling of percent predicted FVC over time following IV idursulfase treatment using data from patients who received ERT for 5 years or more. (**a**) Change in predicted means and 95% CI by age at ERT start (colored lines) and individual patient values (gray lines) from pre-ERT up to 8 years after IV idursulfase start in the main analysis population (patients with data available for at least two time points in total and at least one post-ERT time point; n = 84). (**b**) Predicted means by age at ERT start at pre-ERT and 8 years post-ERT in the main analysis population and the internal validation population (patients with data available at five or more time points). Patients who had cognitive impairment at any time were excluded from the model. Patients aged under 5 years at the time of assessment were also excluded owing to the unreliability of these assessments in children aged under 5 years [17]; pre-ERT values are therefore labeled ‘not applicable’ for patients aged under 5 years at ERT start. Values corresponding to absolute FVC above 10 L were excluded from the model. Abbreviations: CI, confidence interval; ERT, enzyme replacement therapy; FVC, forced vital capacity; IV, intravenous; NA, not applicable; ND, not determined; SE, standard error

**Fig. 4:**
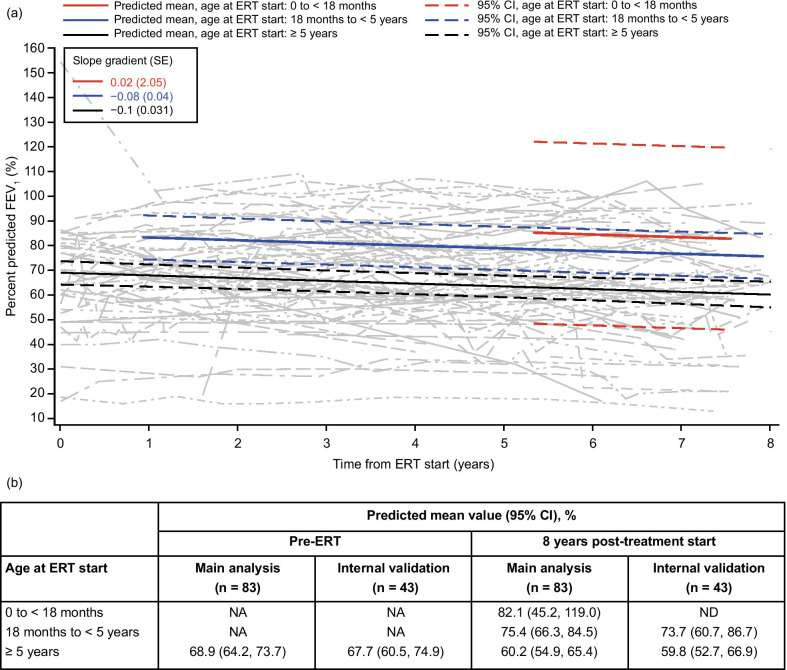
Modeling of percent predicted FEV_1_ over time following IV idursulfase treatment using data from patients who received ERT for 5 years or more. (**a**) Change in predicted means and 95% CIs by age at ERT start (colored lines) and individual patient values (gray lines) from pre-ERT up to 8 years after IV idursulfase start in the main analysis population (patients with data available for at least two time points in total and at least one post-ERT time point; n = 83). (**b**) Predicted means by age at ERT start at pre-ERT and 8 years post-ERT in the main analysis population and the internal validation population (patients with data available at five or more time points). Patients who had cognitive impairment at any time were excluded from the model. Patients aged under 5 years at the time of assessment were also excluded owing to the unreliability of these assessments in children aged under 5 years [17]; pre-ERT values are therefore labeled ‘not applicable’ for patients aged under 5 years at ERT start. Values corresponding to absolute FEV_1_ above 10 L were excluded from the model. Abbreviations: CI, confidence interval; ERT, enzyme replacement therapy; FEV_1_, forced expiratory volume in 1 s; IV, intravenous; NA, not applicable; ND, not determined; SE, standard error

Model-derived percent predicted FVC and FEV_1_ values decreased slightly over time following IV idursulfase treatment in all age groups among patients without cognitive impairment (Figs. 3 and 4). In the FVC model (n = 84), the mean percent predicted value decreased by just over 4 percentage points at 8 years post-ERT start compared with baseline in patients aged ≥ 5 years at treatment start; in the FEV_1_ model (n = 83), the mean percent predicted value decreased by just under 9 percentage points in this age group. In the two groups aged under 5 years at ERT start, values were not modeled until patients reached the age of 5 years. However, at the later time points after ERT start, when patients in all groups had reached an age suitable for the assessments, trends were similar across all ages at treatment start. Patients aged 0 to < 18 months had the highest mean predicted values at 8 years post-ERT start overall, for both percent predicted FVC and percent predicted FEV_1_ (Figs. 3 and 4). Although gradual decreases were observed in percent predicted pulmonary measurements, model-predicted absolute FVC and FEV_1_ increased over time for all ages at ERT start (Additional file 1; Figs S2 and S3).

### 6MWT

**Fig. 5:**
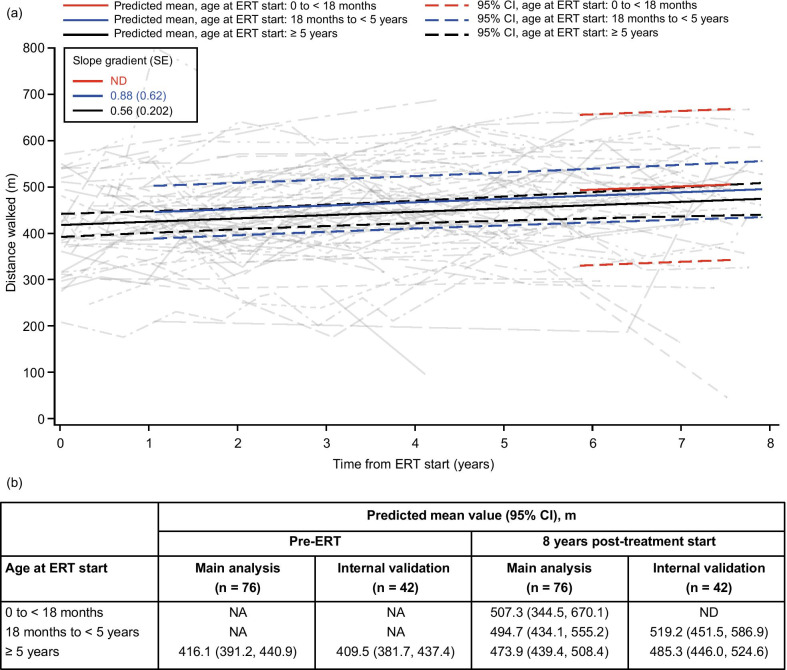
Modeling of distance walked in 6MWT over time following IV idursulfase treatment using data from patients who received ERT for 5 years or more. (**a**) Change in predicted means and 95% CIs by age at ERT start (colored lines) and individual patient values (gray lines) from pre-ERT up to 8 years after IV idursulfase start in the main analysis population (patients with data available for at least two time points in total and at least one post-ERT time point; n = 76). (**b**) Predicted means by age at ERT start at pre-ERT and 8 years post-ERT in the main analysis population and the internal validation population (patients with data available at five or more time points). Patients who had cognitive impairment at any time and patients who needed assistance to perform the assessment were excluded from the model. Patients aged under 5 years at the time of assessment were also excluded owing to the unreliability of these assessments in children aged under 5 years [17]; pre-ERT values are therefore labeled ‘not applicable’ for patients aged under 5 years at ERT start. For reference, the mean (95% CI) distance walked in a previous 6MWT analysis of a population of healthy male individuals increased from 536.5 m (494.1, 578.9) in boys aged 3–5 years to 577.8 m (564.0, 591.6), 672.8 m (656.6, 689.2), 697.8 m (681.2, 714.4) and 725.8 m (709.3, 742.4) in boys aged 6–8 years, 9–11 years and 12–15 years and in adolescent males aged ≥ 16 years, respectively [33]. Abbreviations: 6MWT, 6-min walk test; CI, confidence interval; ERT, enzyme replacement therapy; IV, intravenous; NA, not applicable; ND, not determined; SE, standard error

In total, 76 patients without cognitive impairment were included in the 6MWT model. The distance walked increased gradually over time after treatment in all age groups, with an increase of over 50 m in mean predicted values at 8 years post-ERT compared with baseline in patients aged ≥ 5 years at ERT start (Fig. 5). In the two groups aged under 5 years at ERT start, values were not modeled for patients until they reached the age of 5 years; however, trends at later time points, when patients in all groups had reached an age suitable for the assessment, were similar across all ages at ERT start. Overall, patients aged 0 to < 18 months at ERT start had the highest predicted mean walking distance at 8 years post-ERT start.

### Palpable liver size

**Fig. 6:**
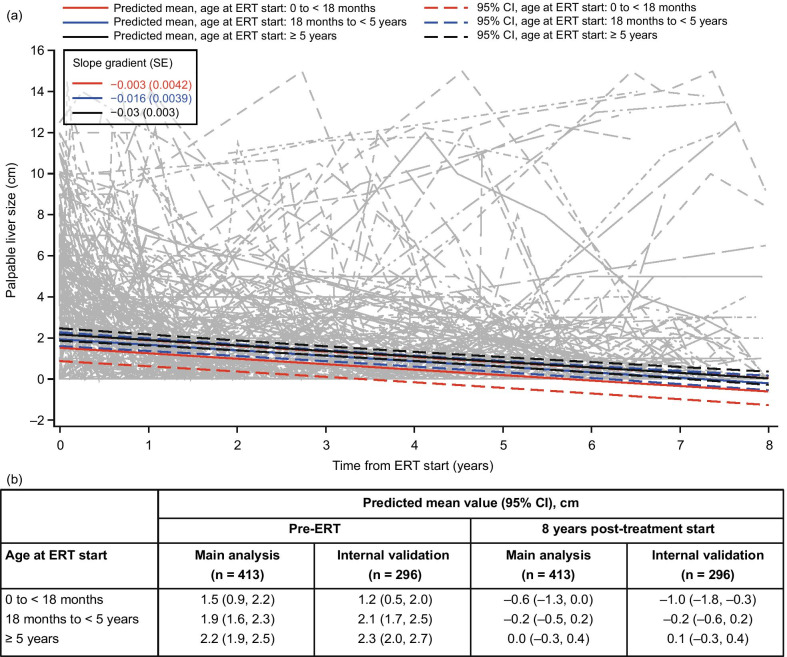
Modeling of palpable liver size over time following IV idursulfase treatment using data from patients who received ERT for 5 years or more. (**a**) Change in predicted means and 95% CIs by age at ERT start (colored lines) and individual patient values (gray lines) from pre-ERT up to 8 years after IV idursulfase start in the main analysis population (patients with data available for at least two time points in total and at least one post-ERT time point; n = 413). (**b**) Predicted means by age at ERT start at pre-ERT and 8 years post-ERT in the main analysis population and the internal validation population (patients with data available at five or more time points). The size of a non-palpable liver was imputed as zero in the model. Abbreviations: CI, confidence interval; ERT, enzyme replacement therapy; IV, intravenous; SE, standard error

Liver size, as estimated by palpation, decreased over time in the model (n = 413) for all three age groups to a similar extent, with a decrease of more than 2 cm in the mean predicted values at 8 years post-ERT compared with baseline in each age group (Fig. 6). The model predicts that, on average, the liver is no longer palpable after 4.4, 7.4 and 8.2 years of treatment for patients who initiated ERT aged < 18 months, 18 months to < 5 years and ≥ 5 years at treatment start, respectively. The liver size values below zero observed after an extended period of treatment are not reflective of real-world observations and are an artifact of the model owing to the fact that many patients had a liver that was no longer palpable post-treatment. In an alternative two-part model, liver size decreased over time in the two age groups included (< 5 years and ≥ 5 years), as in the original model, although the decreases were not as pronounced (Additional file 1; Table S2). In the two-part model, no estimates were below zero.

### Internal validation

An additional analysis was performed for each parameter to provide an internal validation of the model and to assess its robustness, using data from patients with measurements taken at five or more time points. The trends observed in this internal validation were similar to the main analyses for each parameter assessed (Figs. 1b, 2, 3, 4, 5, 6b and Additional file 1; Figs S1b–S3b).

## Discussion

Previous clinical studies and data from real-world analyses have demonstrated that IV ERT with idursulfase is able to stabilize or improve several of the clinical manifestations of MPS II [9–16]. In this study, results from statistical modeling in a diverse population of patients with MPS II indicate that these positive effects on uGAG levels, LVMI, percent predicted FVC and FEV_1_, 6MWT and palpable liver size are predicted to be maintained for a period of at least 8 years following initiation of ERT. The similar results obtained in the associated internal validations suggest that the models provide reliable estimates for all parameters evaluated and support the use of statistical modeling as a valuable additional approach for predicting long-term outcomes in patients with MPS II.

The clinical parameters selected for analysis in this study align with those evaluated in the phase 2/3 clinical trials of IV idursulfase [10, 12] and a later analysis of real-world data from HOS following up to 3 years of IV idursulfase treatment [11]. Overall, our model-predicted findings are broadly consistent with observations from these studies. We observed a continuous decrease in uGAG levels from baseline up to 8 years after ERT initiation, with a similar extent of decline across all ages at ERT start. The predicted percentage decreases of at least 75% after 8 years of ERT are larger than those observed after 1 year of treatment in the initial clinical trial [12] and 3 years of treatment in routine clinical practice [11], suggesting that uGAG levels may continue to decrease over time in patients receiving ERT. It is, however, important to consider the potential confounding influence of natural decreases in uGAG levels over time as children age, which has been observed in patients with MPS II as well as in a control population without MPS II [23, 24].

Also in line with previous real-world observations [11], we observed small decreases in LVMI over time in the overall population, with a predicted stabilization in this parameter for up to 8 years following ERT start. LVMI was also analyzed separately in patients with LVH at baseline, which is often a consequence of cardiac valve disease in patients with MPS II [25, 26]. In patients with versus those without LVH at baseline, the decreases in LVMI over time were significantly larger, which is potentially consistent with a long-term benefit of ERT on cardiac valve function in MPS II. However, the number of patients with LVH was small (n = 19), and it is possible that the decreased LVMI in patients with LVH may reflect a decrease in GAG storage in the walls of the heart rather than, or in addition to, improved cardiac function. The stabilization of LVMI also suggests that long-term ERT may prevent significant valve deterioration, because progressive valve dysfunction would be expected to cause cardiac enlargement in MPS II [25]. However, it is important to note that changes in BSA were not modeled in this study, and the predicted decreases in LVMI are likely to reflect a combined effect of increasing BSA over time and relative stabilization of cardiac mass. It should also be acknowledged that evidence for possible prevention of valve deterioration with long-term ERT appears to be in contrast with some of the findings of a recent long-term study of the impact of ERT in pediatric-onset MPS II [16]. In this study, long-term ERT resolved LVH in all patients with the condition at baseline but, over a follow-up of up to almost 20 years, the prevalence of mitral valve involvement increased by 15% and overall valvular disease progressed in 40% of patients. However, patient numbers were relatively small, with longitudinal data available for 46 patients, and the study was limited to data collected from three centers in a single country (England). By comparison, the current model of LVMI incorporated long-term data for 250 patients across multiple sites and countries. Future studies and analyses are required to establish fully the effects of long-term ERT on valve deterioration in patients with MPS II.

Our findings relating to pulmonary function indicated small decreases in percent predicted FVC and FEV_1_ at 8 years post-ERT start, calculated with reference to the expected values in patients without MPS II of the same sex, age and height [20, 21]. This is broadly in line with previous results from the long-term extension study of IV idursulfase, which found that percent predicted FVC did not change significantly after 2 and 3 years of ERT, despite a small initial improvement after 1 year [10]. This general stabilization in percent predicted values suggests that the deficit in pulmonary function is not worsening over time compared with individuals of a similar age and height without MPS II. In addition, modeled absolute FVC and FEV_1_ values continued to increase continuously for up to 8 years after treatment start, which extends previous observations for absolute (rather than percent predicted) values [10–12]. Together, these findings support a positive effect of IV idursulfase on overall pulmonary function. The lack of increase in percent predicted values may reflect that the formula used in these calculations assumes normal growth and height for age [27], which generally do not apply for patients with MPS II [28]. ERT with IV idursulfase has, however, also been shown to partially ameliorate the short stature associated with MPS II [10, 29, 30], and the potential contribution of relative increases in height to the observed stabilization in percent predicted pulmonary values should also be considered.

The gradual increase observed in 6MWT distance over time supports previous observations [10–12] and suggests that the positive effect of IV idursulfase on walking ability is sustained in the longer term. The increase of approximately 50 m at 8 years post-ERT initiation in patients aged ≥ 5 years at treatment start is slightly higher than the overall median change of 41 m observed after 3 years of IV idursulfase treatment in routine clinical practice [11]. It may, however, be expected that an upper limit of improvement would be reached in the long term sometime after 8 years of treatment, particularly once adulthood and full growth are attained. It is also important to note that 6MWT distance increases in healthy children as they age [31–33]. In one cohort of healthy children and adolescents, the mean 6MWT distance increased from 536.5 m in boys aged 3–5 years to 697.8 m in those aged 12–15 years and 725.8 m in adolescent males aged 16 years or older [33]. Although the increase in 6MWT distance with age may confound the interpretation of the present results, findings from the initial placebo-controlled clinical trial [12], as well as the expected adverse impact of joint manifestations on mobility in untreated patients over longer time periods [34], suggest that the observed improvement is likely to be largely treatment-related.

An enlarged liver as a result of GAG storage is a common clinical manifestation of MPS II [3, 35]. Consistent with previous findings [10–12], we observed a continual decline in liver size up to 8 years post-ERT start, with the liver no longer being palpable in many patients. An important limitation of this model is that prediction of palpable liver sizes below zero does not reflect observations in real-world clinical practice, in which the minimum recordable size would be zero (‘non-palpable’). In the alternative two-part model, the estimates did not fall below zero; results from this model also showed a continual decline in liver size up to 8 years post-ERT start, although the decreases were less pronounced than in the original model. In addition, it should be noted that liver measurements obtained via palpation may be influenced by variable lung inflation, and the positioning of the liver under the rib cage may also necessitate percussion to identify the upper and/or lower border accurately. Although the use of palpation to estimate liver size is standard in the management of patients with MPS II, abdominal ultrasound and/or magnetic resonance imaging would provide more accurate measurements [11, 12, 22]. Nonetheless, the models indicate that ERT-associated decreases in liver size may continue in the long term. Importantly, the results also indicate the average time at which a liver may be expected to be no longer palpable following treatment (varying from just over 4 years in patients aged < 18 months at treatment start to just over 8 years in those aged ≥ 5 years at treatment start).

Overall, these results suggest that the benefits associated with IV idursulfase treatment observed in the initial clinical trials [10, 12] and after 3 years of treatment in routine clinical practice [11] are maintained in the longer term. The results are also in line with observations in localized studies of smaller numbers of IV idursulfase-treated patients, including one with 10 years of follow-up [13–16], and provide valuable support for these findings in a larger and more diverse population of patients with MPS II.

We also assessed the effects of IV ERT on long-term clinical outcomes in different patient categories defined by age at the start of treatment. It has previously been suggested, based on case series and clinical experience from MPS II and other LSDs, that initiation of treatment at a presymptomatic or early symptomatic stage is likely to be associated with improved outcomes [36–39]. In this study, we found that predicted changes over time in clinical parameters generally appeared to be similar in the groups of patients stratified by age at ERT start, suggesting that IV ERT with idursulfase may stabilize or improve somatic manifestations of MPS II regardless of when treatment is initiated. However, overall, the predicted absolute outcomes for each parameter after 8 years of treatment were most favorable in children starting treatment before the age of 18 months. This largely reflects the fact that patients who were older at treatment start tended to have more severe disease at baseline, as indicated by, for example, larger palpable liver size and higher LVMI. This model and analysis were not designed to compare directly numerical values at specific ages. However, it was apparent that, for all parameters, predicted means after 5–8 years of ERT in those aged < 18 months at ERT start were generally more favorable than the baseline means in patients aged ≥ 5 years at ERT start. Overall, the findings from this study provide further support for the clinical benefits of initiating ERT as early as possible in the disease course in patients with MPS II.

Although this study provides valuable information on the predicted long-term effects of IV idursulfase, there are several limitations that should be considered. First, the findings are descriptive only, and the nature of the modeling approach meant that it was not possible to include an appropriate untreated comparator group. It is therefore not possible to identify statistically significant trends or differences between groups. The models also did not account for any interruptions to ERT during the follow-up period, which may have an impact on overall clinical outcomes over time. Additionally, there may be some bias in the predicted values because the availability of long-term data may be associated with severity of disease. Patients with a more severe disease presentation, who are more likely to die at a younger age, may have fewer long-term data available. Indeed, patients in this analysis with cognitive impairment were found to participate in HOS for a shorter period than those without cognitive impairment. The bias could also potentially operate in the opposite direction, because patients who experience better clinical outcomes and fewer MPS II-related complications may be expected to be less likely to attend regular follow-up visits. However, in the current analysis, there was no evidence of fewer follow-up visits in patients with no cognitive impairment compared with those with cognitive impairment, suggesting this direction of bias did not play a major role. It should also be noted that the models for FVC, FEV_1_ and 6MWT excluded patients with cognitive impairment, owing to the unsuitability of the assessments in these patients, potentially limiting the applicability of results from these models to those patients without cognitive impairment and possibly with a milder phenotype. To increase the amount of data available for model development, patients were not required to have a baseline measurement available for the relevant parameter to be included. One limitation arising from this model design is that the patients with post-ERT data available may not be the same individuals as those with baseline data available owing to the nature of long-term follow-up, with the frequency of clinic visits and assessments likely to differ between patients. Therefore, although the findings as a whole give insight into predicted long-term trends for each parameter, reported values at individual time points should be considered as cross-sectional predictions. The internal validation for each parameter suggested that limitations in data completeness did not have a large impact on the model results; however, model robustness would ideally be fully evaluated via testing with an independent data set.

Other limitations are associated with the HOS registry as a source of data for the model. Information in HOS is captured during routine clinical practice and, as such, the methods and techniques used for assessments and assays are not standardized across different sites. In particular, uGAG levels may be measured using varying methodologies at different laboratories, and cognitive impairment status may be based on the clinical impression of each physician and/or standardized cognitive tests. The amount of data used in the 6MWT and pulmonary function models was also limited by the exclusion of patients with cognitive impairment and those aged < 5 years, which was necessary owing to the unsuitability of these assessments for these patient groups. In addition, although multilevel mixed modeling is generally employed for assessing longitudinal data collected from multiple centers, it was not possible in the current analysis because of the large number of centers involved (approximately 130).

Despite these limitations, the approach used in this study overcomes some of the challenges associated with the evaluation of long-term outcomes for patients with MPS II. The collection of complete, long-term data for patients enrolled in registries can be challenging owing to the voluntary nature of assessments and data entry [17] and, for rare genetic diseases such as MPS II, the opportunities to collect large amounts of data over long periods of time are particularly limited. Statistical modeling therefore represents a valuable additional approach for the evaluation of real-world clinical outcomes in these patient populations.

## Conclusions

In summary, these results provide novel predictions of the expected trends in clinical and biochemical parameters for up to 8 years following the start of ERT and extend the existing evidence that long-term IV idursulfase has a positive effect on uGAG levels, LVMI, percent predicted FVC and FEV_1_, 6MWT and liver size in patients with MPS II. They also provide support for the benefit of IV idursulfase on somatic manifestations of MPS II even when initiated later in the disease course, although the most favorable overall outcomes are likely to be observed in patients starting ERT before the age of 18 months. In view of the fact that idursulfase only became available as an approved therapy 13 years ago, and given that many patients will not have started ERT until some years after this initial approval, these 8-year modeling data based on a large and diverse patient group represent a considerable proportion of the overall clinical experience with idursulfase. These findings highlight the value of statistical modeling to evaluate clinical outcomes when the available data are limited, and similar approaches are likely to be useful for further analyses in MPS II and other rare diseases in the future.

## Supplementary Information

**Additional file 1**. **Table S1** Summary of demographics and clinical characteristics for patients included in at least one statistical model. **Table S2** Comparison of results from two modeling approaches to evaluate palpable liver size over time following IV idursulfase treatment using data from patients who received ERT for 5 years or more. (**a**) Results from linear mixed-effects model. (**b**) Results from two-part hurdle Poisson mixed-effects model. Different age groups were used in the two-part model owing to limitations of this modeling approach. **Fig. S1** Modeling of LVMI over time following IV idursulfase treatment in patients with LVH at baseline using data from patients who received ERT for 5 years or more. **Fig. S2** Modeling of absolute FVC over time following IV idursulfase treatment using data from patients who received ERT for 5 years or more. **Fig. S3** Modeling of absolute FEV_1_ over time following IV idursulfase treatment using data from patients who received ERT for 5 years or more.

## Data Availability

The data sets, including redacted study protocol, redacted statistical analysis plan, and individual participants' data supporting the results reported in this article, will be made available within 3 months from initial request, to researchers who provide a methodologically sound proposal. The data will be provided after its deidentification in compliance with applicable privacy laws, data protection and requirements for consent and anonymization.

## Abbreviations

6MWT: 6-min walk test
BMT: Bone marrow transplant
BSA: Body surface area
CI: Confidence interval
ERT: Enzyme replacement therapy
FEV_1_: Forced expiratory volume in 1 s
FVC: Forced vital capacity
GAG: Glycosaminoglycan
HOS: Hunter Outcome Survey
I2S: Iduronate-2-sulfatase
IV: Intravenous
LSD: Lysosomal storage disease
LVH: Left ventricular hypertrophy
LVMI: Left ventricular mass index
MPS II: Mucopolysaccharidosis II
SE: Standard error
uGAG: Urinary glycosaminoglycan
