# Supplementary material for: Evaluation of the long-term treatment effects of intravenous idursulfase in patients with mucopolysaccharidosis II (MPS II) using statistical modeling: data from the Hunter Outcome Survey (HOS)
Source: Orphanet J Rare Dis. 2021 Oct 30;16:456. doi: 10.1186/s13023-021-02052-4 (PMC8557006; doi:10.1186/s13023-021-02052-4)
Supplement: Supplementary file 1 — Additional file 1. Table S1 Summary of demographics and clinical characteristics for patients included in at least one statistical model. Table S2 Comparison of results from two modeling approaches to evaluate palpable liver size over time following IV idursulfase treatment using data from patients who received ERT for 5 years or more. (a) Results from linear mixed-effects model. (b) Results from two-part hurdle Poisson mixed-effects model. Different age groups were used in the two-part model owing to limitations of this modeling approach. Fig. S1 Modeling of LVMI over time following IV idursulfase treatment in patients with LVH at baseline using data from patients who received ERT for 5 years or more. Fig. S2 Modeling of absolute FVC over time following IV idursulfase treatment using data from patients who received ERT for 5 years or more. Fig. S3 Modeling of absolute FEV1 over time following IV idursulfase treatment using data from patients who received ERT for 5 years or more. [file 13023_2021_2052_MOESM1_ESM.pdf]

Additional File 1

Table S1 Summary of demographics and clinical characteristics for patients included in at least one statistical model

Characteristic	Patients (N = 481)
Region, n (%)	
Europe[a]	277 (57.6)
North America[b]	131 (27.2)
Latin America[c]	56 (11.6)
Asia Pacific[d]	17 (3.5)
Age at onset of symptoms, years (n = 413)	
Median (P10; P90)	1.5 (0.3; 4.2)
Age at diagnosis, years (n = 465)	
Median (P10; P90)	3.3 (1.0; 6.9)
Age at ERT start, years	
Median (P10; P90)	5.5 (2.0; 17.4)
Age at HOS entry, years	
Median (P10; P90)	6.9 (2.5; 19.0)
Age at last visit, years	
Median (P10; P90)	15.0 (9.7; 27.5)
Duration in HOS, years	
Median (P10; P90)	8.1 (3.0; 11.9)
Cognitive impairment at any time, n (%) (n = 479)	319 (66.6)

[a]Europe includes patients from Austria, Belgium, Bulgaria, Croatia, Czech Republic, Denmark, France, Germany, Greece, Hungary, Ireland, Italy, the Netherlands, Norway, Poland, Portugal, Romania, Russia, Spain, Sweden, Switzerland and the UK; [b]North America includes patients from Canada and the USA; [c]Latin America includes patients from Argentina, Brazil, Colombia and Venezuela; [d]Asia Pacific includes patients from Taiwan. Data were not available for all patients for some characteristics; overall n numbers are specified next to these characteristics (n = 481 unless otherwise stated).

Abbreviations: ERT, enzyme replacement therapy; HOS, Hunter Outcome Survey; P10, 10th percentile; P90, 90th percentile

Table S2 Comparison of results from two modeling approaches to evaluate palpable liver size over time following IV idursulfase treatment using data from patients who received ERT for 5 years or more. (**a**) Results from linear mixed-effects model. (**b**) Results from two-part hurdle Poisson mixed-effects model. Different age groups were used in the two-part model owing to limitations of this modeling approach

(a)

Time from ERT start, years	Predicted mean value (95% CI), cm		
	Age at ERT start: 0 to < 18 months	Age at ERT start: 18 months to < 5 years	Age at ERT start: ≥ 5 years
0	1.5 (0.9, 2.2)	1.9 (1.6, 2.3)	2.2 (1.9, 2.5)
1	1.3 (0.6, 1.9)	1.7 (1.4, 2.0)	1.9 (1.6, 2.2)
2	1.0 (0.4, 1.6)	1.4 (1.1, 1.7)	1.6 (1.4, 1.9)
3	0.7 (0.1, 1.3)	1.1 (0.9, 1.4)	1.4 (1.1, 1.6)
4	0.5 (–0.2, 1.1)	0.9 (0.6, 1.1)	1.1 (0.9, 1.3)
5	0.2 (–0.4, 0.8)	0.6 (0.3, 0.9)	0.8 (0.6, 1.1)
6	–0.1 (–0.7, 0.5)	0.3 (0.0, 0.6)	0.6 (0.3, 0.8)
7	–0.3 (–1.0, 0.3)	0.1 (–0.2, 0.4)	0.3 (0.0, 0.6)
8	–0.6 (–1.3, 0.0)	–0.2 (–0.5, 0.2)	0.0 (–0.3, 0.4)

(b)

Time from ERT start, years	Predicted mean value (95% CI), cm	
	Age at ERT start: 0 to < 5 years	Age at ERT start: ≥ 5 years
0	1.6	3.1
1	1.5 (1.5, 1.5)	2.7 (2.7, 2.8)
2	1.5 (1.5, 1.5)	2.3 (2.2, 2.3)
3	1.4 (1.4, 1.4)	1.9 (1.9, 1.9)
4	1.3 (1.3, 1.4)	1.7 (1.7, 1.7)
5	1.3 (1.3, 1.3)	1.5 (1.5, 1.5)
6	1.3 (1.3, 1.3)	1.4 (1.4, 1.4)
7	1.2 (1.2, 1.2)	1.3 (1.3, 1.3)
8	1.2 (1.2, 1.2)	1.2 (1.2, 1.2)

Fig. S1 Modeling of LVMI over time following IV idursulfase treatment in patients with LVH at baseline using data from patients who received ERT for 5 years or more. (**a**) Change in predicted means and 95% CIs by age at ERT start (colored lines) and individual patient values (gray lines) from pre-ERT up to 8 years after IV idursulfase start in the main analysis population (patients with data available for at least two time points in total and at least one post-ERT time point; n = 19). (**b**) Predicted means by age at ERT start at pre-ERT and 8 years post-ERT in the main analysis population and the internal validation population (patients with data available at five or more time points). LVMI values > 400 g/m^2 were excluded from the model. LVH was defined as LVMI > 102 g/m^2. For reference, 50–102 g/m^2 (indexed to BSA) has previously been defined as a normal LVMI range in male patients [19].

Abbreviations: BSA, body surface area; CI, confidence interval; ERT, enzyme replacement therapy; IV, intravenous; LVH, left ventricular hypertrophy; LVMI, left ventricular mass index; ND, not determined; SE, standard error

(a)

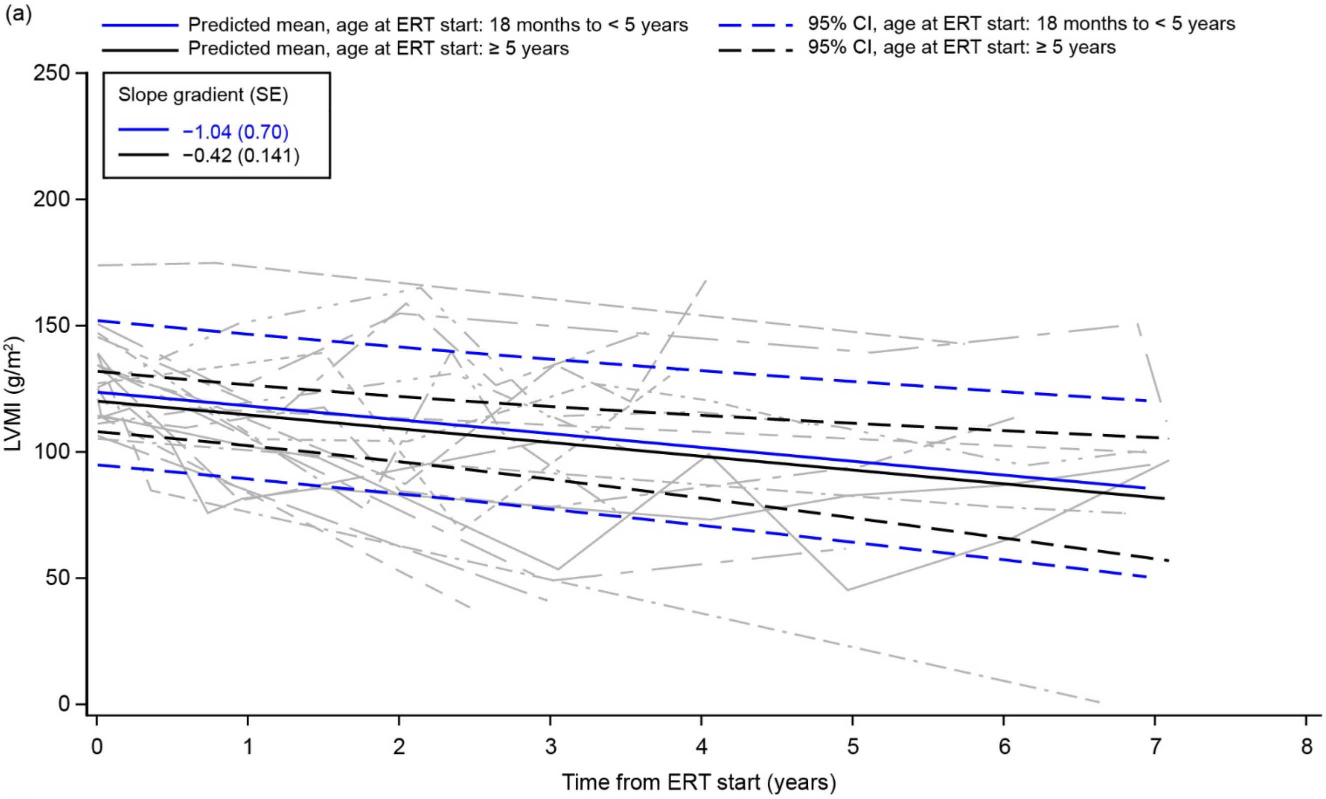

(b)

	Predicted mean value (95% CI), g/m^2			
	Pre-ERT		8 years post-treatment start	
**Age at ERT start**	**Main analysis (n = 19)**	**Internal validation (n = 9)**	**Main analysis (n = 19)**	**Internal validation (n = 9)**
0 to < 18 months	ND	ND	ND	ND
18 months to < 5 years	123.6 (94.9, 152.2)	147.2 (98.5, 195.8)	79.7 (42.7, 116.7)	123.8 (71.2, 176.5)
≥ 5 years	120.1 (108.1, 132.2)	119.5 (103.5, 135.4)	76.3 (49.4, 103.1)	96.1 (68.6, 123.6)

Fig. S2 Modeling of absolute FVC over time following IV idursulfase treatment using data from patients who received ERT for 5 years or more. (**a**) Change in predicted means and 95% CIs by age at ERT start (colored lines) and individual patient values (gray lines) from pre-ERT up to 8 years after IV idursulfase start in the main analysis population (patients with data available for at least two time points in total and at least one post-ERT time point; n = 86). (**b**) Predicted means by age at ERT start at pre-ERT and 8 years post-ERT in the main analysis population and the internal validation population (patients with data available at five or more time points). Patients who had cognitive impairment at any time were excluded from the model. Patients aged under 5 years at the time of assessment were also excluded owing to the unreliability of these assessments in children aged under 5 years [17]; pre-ERT values are therefore labeled 'not applicable' for patients aged under 5 years at ERT start. FVC values above 10 L were excluded from the model.

Abbreviations: CI, confidence interval; ERT, enzyme replacement therapy; FVC, forced vital capacity; IV, intravenous; NA, not applicable; ND, not determined; SE, standard error

(a)

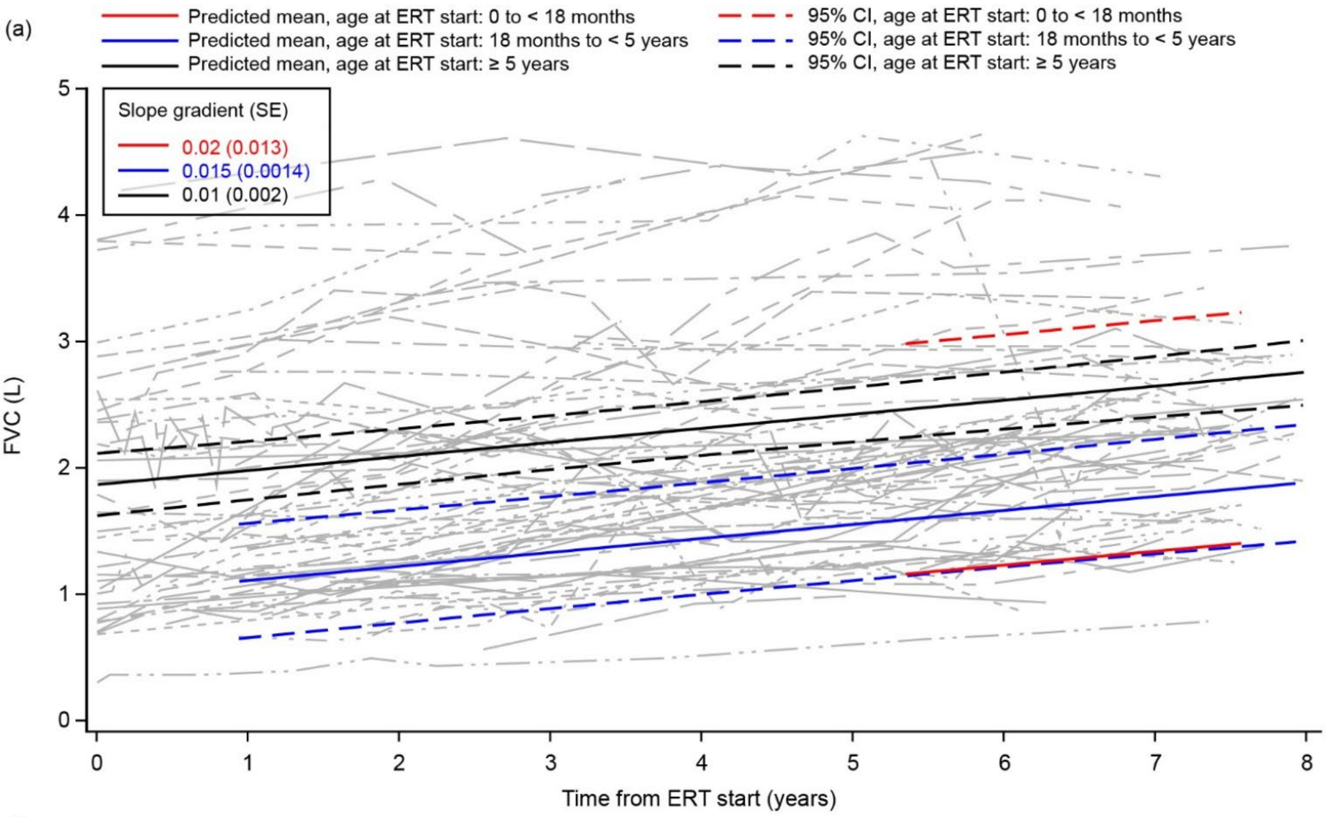

(b)

	Predicted mean value (95% CI), L			
	Pre-ERT		8 years post-treatment start	
Age at ERT start	Main analysis (n = 86)	Internal validation (n = 42)	Main analysis (n = 86)	Internal validation (n = 42)
0 to < 18 months	NA	NA	1.5 (–0.4, 3.3)	ND
18 months to < 5 years	NA	NA	1.9 (1.4, 2.3)	1.7 (1.2, 2.2)
≥ 5 years	1.9 (1.6, 2.1)	1.6 (1.4, 1.9)	2.8 (2.5, 3.0)	2.5 (2.2, 2.8)

Fig. S3 Modeling of absolute FEV_1 over time following IV idursulfase treatment using data from patients who received ERT for 5 years or more. (**a**) Change in predicted means and 95% CIs by age at ERT start (colored lines) and individual patient values (gray lines) from pre-ERT up to 8 years after IV idursulfase start in the main analysis population (patients with data available for at least two time points in total and at least one post-ERT time point; n = 86). (**b**) Predicted means by age at ERT start at pre-ERT and 8 years post-ERT in the main analysis population and the internal validation population (patients with data available at five or more time points). Patients who had cognitive impairment at any time were excluded from the model. Patients aged under 5 years at the time of assessment were also excluded owing to the unreliability of these assessments in children aged under 5 years [17]; pre-ERT values are therefore labeled 'not applicable' for patients aged under 5 years at ERT start. FEV_1 values above 10 L were excluded from the model.

Abbreviations: CI, confidence interval; ERT, enzyme replacement therapy; FEV_1, forced expiratory volume in 1 second; IV, intravenous; NA, not applicable; ND, not determined; SE, standard error

(a)

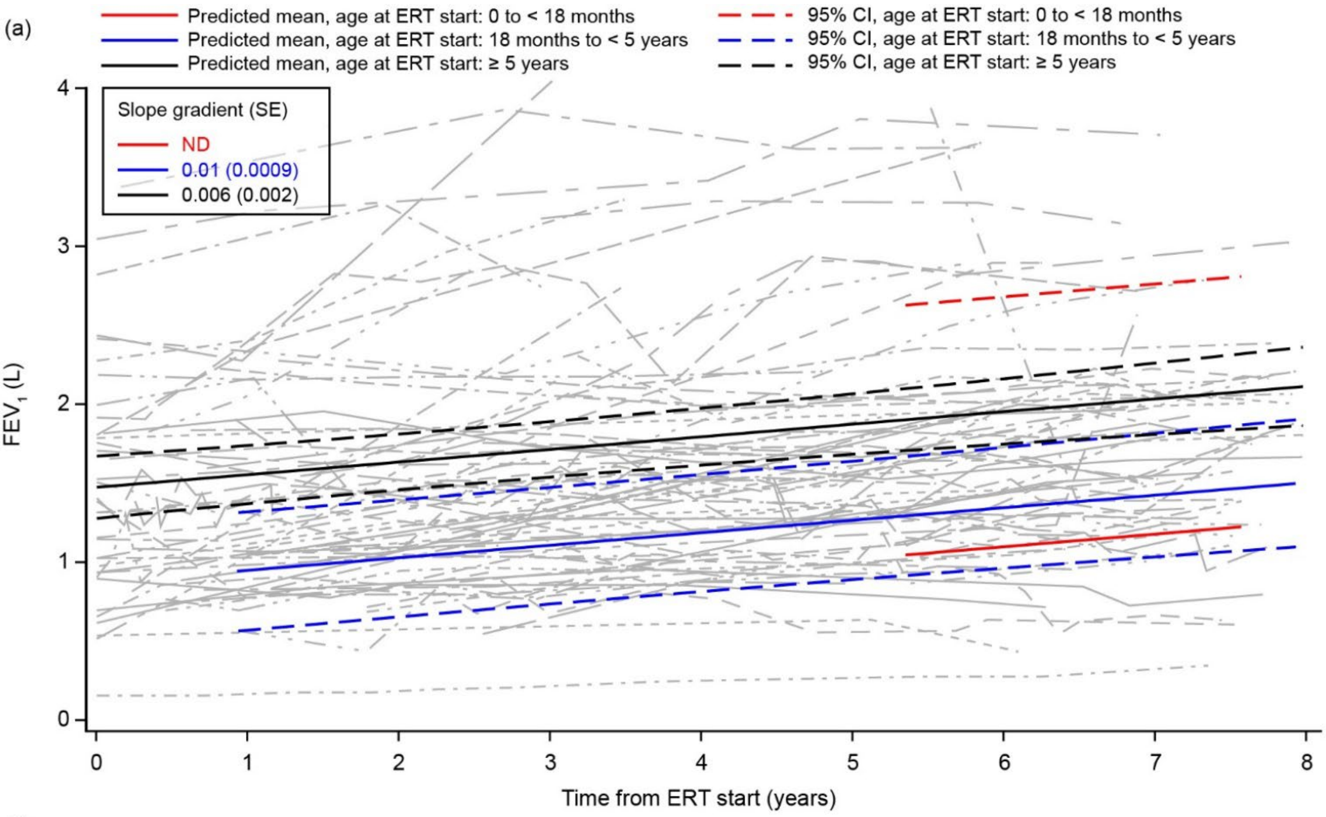

(b)

	Predicted mean value (95% CI), L			
	Pre-ERT		8 years post-treatment start	
**Age at ERT start**	**Main analysis (n = 86)**	**Internal validation (n = 43)**	**Main analysis (n = 86)**	**Internal validation (n = 43)**
0 to < 18 months	NA	NA	1.3 (–0.3, 2.8)	ND
18 months to < 5 years	NA	NA	1.5 (1.1, 1.9)	1.4 (1.0, 1.8)
≥ 5 years	1.5 (1.3, 1.7)	1.3 (1.1, 1.5)	2.1 (1.9, 2.4)	1.9 (1.6, 2.1)
